# Retractions of publications on central nervous system tumors based on the Retraction Watch Database

**DOI:** 10.1186/s41016-026-00444-8

**Published:** 2026-08-10

**Authors:** Xiaoyan Gao, Renjie Mao, Fa Lin, Jing Shang, Jingjing Wu, Lu Chen, Hao Wang

**Affiliations:** 1 Department of Science and Technology, Beijing Tiantan Hospital, Capital Medical University, Beijing, China; 2 Department of Neurosurgery, Beijing Tiantan Hospital, Capital Medical University, Beijing, China

**Keywords:** Central nervous system neoplasms, Publication retraction, Research misconduct, Bibliometrics

## Abstract

**Background:**

Retraction serves as a critical mechanism for correcting flawed publications and preserving the integrity of the scientific literature. Systematic analysis focused on retractions in Central Nervous System (CNS) tumor research is still lacking.

This study characterized retracted CNS tumor publications regarding temporal trends, geographic distribution, retraction reasons, citation patterns, and retraction lag.

**Methodology:**

Retraction data were downloaded from the Crossref/Retraction Watch Database on December 31, 2025. CNS tumor‑related retractions were identified using tumor terms derived from the 2021 WHO Classification of Tumors of the Central Nervous System. Eligible records were screened manually, and tumor type, article type, retraction reason, first-author country, author count and time to retraction were extracted. Citation data were retrieved from Web of Science, and journal impact factor (JIF) and quartile were obtained from the 2024 Journal Citation Reports. Descriptive statistics, the Mann–Kendall test and Kruskal‑Wallis test were used.

**Results:**

582 CNS tumor articles were included. CNS tumor retractions increased over time and peaked in 2023. "Gliomas, glioneuronal tumors, and neuronal tumors" accounted for 74.57% (434/582). 80.58% (469/582) were retracted due to misconduct. In terms of absolute numbers, China was the leading first‑author country, followed by USA and India. Basic research accounted for 75.26% (438/582). The median time to retraction was 805.5 days (IQR: 423.25–1,711 days). A total of 515 articles received at least one citation (total citations 12,151), and 89.71% (462/515) had citation after retraction. The median pre‑retraction, post‑retraction, and total citations were 5 (IQR: 1–17), 3 (IQR: 1–7), and 10 (IQR: 3–24), respectively. Significant differences were observed in normalized citation counts and retraction lag across most retraction reason categories, JIF and quartiles, article types, author counts, and first‑author countries.

**Conclusions:**

CNS tumor retractions have increased markedly as the primary driver. The persistent post-retraction citation of flawed research highlights a critical gap threatening evidence-based neuro-oncology.

**Supplementary Information:**

The online version contains supplementary material available at 10.1186/s41016-026-00444-8.

## Background

Retraction is a critical mechanism for correcting erroneous or flawed published findings and safeguarding the integrity of the academic ecosystem. By retracting flawed scholarly articles, the scientific community can prevent their continued dissemination and citation, thereby reducing potential adverse effects on both basic research and clinical decision making [1, 2]. In recent years, driven by the rapid growth of global research output, the annual number of retractions has steadily increased. Moreover, the predominant reasons for retraction have shifted from traditional forms of misconduct (plagiarism and duplicate publication) to more covert academic misconduct, including data manipulation, image fabrication, and peer review problems [3–5].

The Retraction Watch Database (RWD), established by Adam Marcus and Ivan Oransky in 2010, has become an internationally recognized source of retraction data. In 2023, Crossref acquired RWD and made it publicly accessible [6], providing a comprehensive resource for bibliometric analyses. Previous studies have demonstrated that RWD outperforms PubMed and Web of Science in identifying retracted publications in the biomedical literature, making it a valuable tool for bibliometric analyses of retractions across various research fields [7]. Retraction analyses based on RWD have been conducted in many medical fields [5, 8–12] . Notably, retracted papers related to nervous system tumors have emerged repeatedly. For example, a study published in Cell in 2014 was retracted in 2017 because the authors could not replicate their original core findings [13]. In another instance, a pharmacodynamic study on anlotinib for the treatment of high-grade gliomas was retracted by the publisher due to compromise of the peer review process [14]. Despite these concerning cases, there remains a lack of systematic analysis on retracted publications of central nervous system tumors. In the present study, we applied RWD as the primary data sources and systematically analyzed the overall characteristics of retracted papers in this field, including temporal and geographic distribution, reasons for retraction, article type, citation counts, journal and publisher profiles as well as the retraction lag, etc. Given the rising trend of retractions in biomedical research and the absence of systematic data for CNS tumors retractions, this study was conducted to characterize the landscape of retracted publications and inform efforts to maintain research integrity in neuro-oncology.

## Methodology

### Search strategy and data source

Retracted information was downloaded from the Crossref/Retraction Watch database on December 31, 2025. Referring to the 2021 WHO Classification of Tumors of the Central Nervous System: a summary (5th edition) [15], keywords based on central nervous system tumor names were used to search and screen the titles of retracted papers by researchers (X.G. and R.M.) and a preliminary dataset was generated. A complete list of search terms is provided in Additional file 1.

### Inclusion and exclusion criteria

Retraction Nature was limited to "Retraction", and conference papers, meeting abstracts, expressions of concern, reinstatements, and corrections were excluded. Retractions because of the reason "withdrawn (out of date)" were removed because this kind of retraction was part of the routine journal process of update.

### Study selection process

Retractions were further manually reviewed through title and/or abstract by researchers (X.G. and R.M.) to exclude unrelated research of central nervous system tumor. Inter-reviewer agreement was excellent (Cohen's kappa = 0.93, 95% CI: 0.87–0.99). Articles of unclear relevance were adjudicated by a neurosurgeon (F.L.) A final dataset for subsequent analysis was constructed.

### Operational definitions

#### Classification of CNS tumors

Tumor type was categorized manually for each according to the 2021 WHO Classification of Tumors of the Central Nervous System [15]. The classification was based on the tumor entity described in the title, abstract, full text when available, journal metadata, and retraction notice. Two reviewers (X.G. and R.M.) independently screened the records and assigned tumor categories using a prespecified coding framework. For articles without a specified pathological classification, the category "unspecified" was assigned. Articles involving multiple tumor categories were labeled as "mixed" unless one tumor entity was clearly the primary focus. Interreviewer agreement was excellent (Cohen's kappa = 0.90, 95% CI: 0.86–0.94). Disagreements were resolved by discussion; unresolved discrepancies were adjudicated by a third reviewer (F.L.).

#### Classification of article types

Retractions were classified into four categories based on their content and study type by researchers (X.G. and F.L.): Basic research (mechanistic studies, non-human subject research, or research not yet directly applied to clinical practice), Clinical research (case reports, studies involving human participants, or studies focusing on therapeutic or clinical outcomes), Commentary articles, and Review/Meta-analysis articles. Inter-reviewer agreement was excellent (Cohen's kappa = 0.89, 95% CI: 0.85–0.93). Disagreements were resolved through discussion, and unresolved discrepancies were adjudicated by a third reviewer.

#### Classification of retraction reasons

Reasons for retraction were obtained from the Crossref/Retraction Watch database (RWD) according to the category definitions provided in the Retraction Watch Database User Guide [16]. Each retracted article may contain multiple retraction reasons. Referring to the COPE retraction guidelines [2] and the "Rules for the Investigation and Handling of Research Misconduct" issued by the Ministry of Science and Technology of China in 2022 [17], reasons for retraction were re-classified into four broader categories: "Misconduct", "Unverified misconduct", "Error", and "Other" after thorough discussion among the research team. By the following criteria, each retracted article was further identified into any of the four categories by researchers (X.G. and R.M.): if the retraction reasons from RWD contained any reason falling under the "Misconduct" category, the article was defined as retracted due to "Misconduct"; among the remaining articles, if the retraction reasons from RWD contained any reason falling under the "Unverified misconduct" category, the article was defined as retracted due to "Unverified misconduct"; articles whose retraction reasons only included those categorized as "Error" were defined as retracted due to "Error"; all other articles were defined as retracted due to "Other" reasons. Computer-Aided Content or Computer-Generated Content was classified as "Misconduct" referring the recent study [8]. Two researchers independently classified all retractions with complete agreement.

#### Country classification

Articles with affiliations from two or more countries are classified as internationally collaborative, and the first author's country is designated as the country of origin of the retracted article. In the country-level analysis, retracted articles with missing country information in the database were excluded.

#### Journal impact metrics

Clarivate Journal Citation Reports (JCR) was used to obtain journal information, the 2024 journal impact factor (JIF) and JIF quartile [18]. The last reported JIF was used for journals no longer indexed (dropped or suspended) by Clarivate. Articles from journals that had never been indexed were excluded during the analysis of JIF and quartiles.

#### Citation data

Web of Science was used to retrieve citation data of retracted articles. The citation counts were further divided into pre-retraction and post-retraction citations according to the retraction date. Citation analysis was conducted only on articles that could be retrieved in the Web of Science. For further analysis, pre-retraction and post-retraction citation counts were normalized using annual pre-retraction citations and annual post-retraction citations. The normalization process was as follows: annual pre-retraction citations = pre-retraction citations/years from publication to retraction; annual post-retraction citations = post-retraction citations/years from retraction to citation data collection (April 12, 2026); annual total retraction citations = total retraction citations/years from publication to citation data collection (April 12, 2026).

#### Time to retraction

Time to retraction (in days) was calculated as the interval between the retraction date and original publication date.

### Data analysis

Descriptive statistics were calculated for the number of authors of each article, as well as the JIF and quartile of the publishing journal. For further analyses, the JIF was grouped as ≤ 3, between 3 and 5 (exclusive), and ≥ 5, while the number of authors was divided into four categories: 1–3, 4–6, 7–9, and ≥ 10.

The annual number of retracted articles was plotted separately by retraction year and publication year. A two-sided Mann–Kendall test was applied to evaluate the significance of any monotonic trend. To quantify the direction and strength of the association with time (retraction year and publication year), Kendall’s τ coefficient along with its 95% confidence interval was derived. By definition, τ values of − 1, 0 and 1 correspond to a perfectly negative, null, and perfectly positive association, respectively.

For continuous variables, time to retraction (days), citation counts, data were presented as median with interquartile range (IQR). Associations between these continuous outcomes and categorical variables (categories of retraction reason, JIF group, JIF quartile, article type, first author’s country, and number of authors group) were evaluated using the Kruskal–Wallis H test. Pairwise comparisons were performed using Bonferroni correction for citation comparisons (pre-retraction vs post-retraction, pre-retraction vs total, and post-retraction vs total) and time to retraction comparisons among subgroups. No global correction was applied across all Kruskal–Wallis tests as each test addressed a distinct research question.

To examine the relationship between time to retraction and first-author country, we analyzed the distribution of original publication years by country and calculated Spearman correlation coefficients between publication year and time to retraction.

*P* values less than 0.05 were considered statistically significant for all analyses. All statistical analyses were performed using SAS version 9.4 (SAS Institute, Cary, NC, USA).

## Results

A total of 68,154 retraction records were retrieved from RWD. After screening and exclusion, 582 retracted CNS tumors articles were included in the study (Fig. 1). The earliest retracted article was published in 1975 while the earliest retraction occurred in 1977. The number of retractions increased in recent years and peaked at 130 in 2023. The number of retracted publications exceeded 30 in 2015 and peaked at 82 in 2022. Notably, 78.01% (454/582) retracted articles were originally published after 2015 (Fig. 2). The annual distribution of publications and retractions is detailed in Additional file 2.The number of retracted publications (τ = 0.69; 95% CI, 0.57–0.81; *P* < 0.001) and retractions (τ = 0.72; 95% CI, 0.58–0.85; *P* < 0.001) exhibited a strong upward trend over time.

**Fig. 1:**
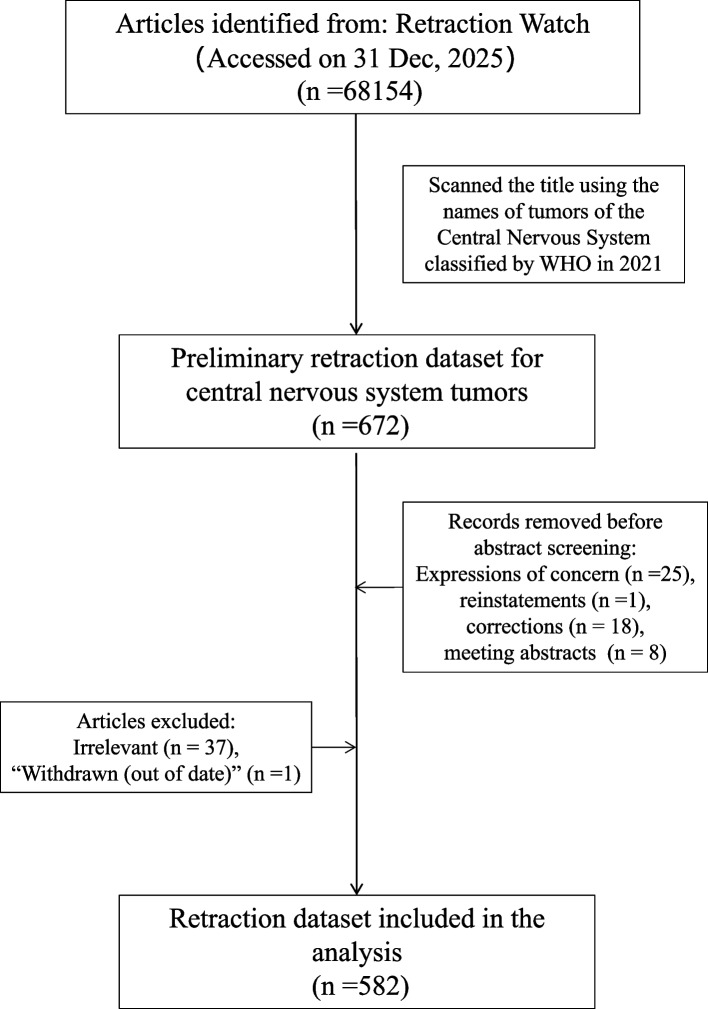
Establishment of the retraction dataset for analysis

**Fig. 2:**
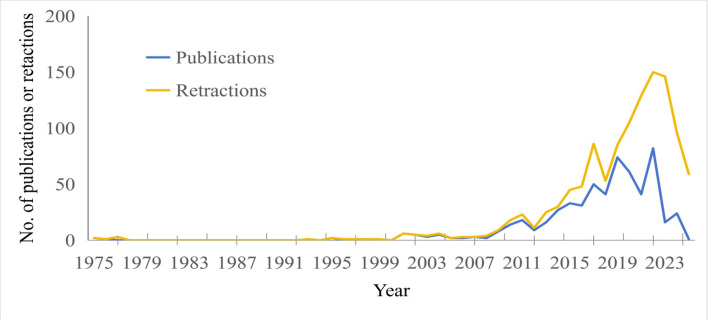
Temporal trends of CNS tumor retractions

Characteristics of CNS tumor retractions are presented in Table 1. Among the 12 categories of central nervous system tumors, retracted papers involved 10 categories. The category "Gliomas, glioneuronal tumors, and neuronal tumors" had the highest number of retractions, with 434 papers and accounted for 74.57% (434/582). Seventy-six distinct retraction reasons were involved in retracted papers. The most common reason was "Investigation by Journal/Publisher", accounting for 47.42% (276/582). These 76 reasons were further classified into four groups: 30 were categorized as "Misconduct", 25 as "Unverified misconduct", 9 as "errors", and 12 as "others" (Table 2). In total, 469 articles were retracted due to misconduct reasons, accounting for 80.58% (469/582). "Paper Mill","Duplication of/in Image" and "Compromised Peer Review" were top 3 "Misconduct" reasons, accounting for 30.58% (178/582), 28.35% (165/582), and 13.57% (79/582), respectively.

**Table 1:** Characteristics of CNS tumor retractions

Characteristic	Number of retractions (%)
Classification of CNS Tumors (*N* = 582)	
Gliomas, glioneuronal tumors, and neuronal tumors	434(74.57%)
Tumors of the sellar region	32(5.50%)
Embryonal tumors	18(3.09%)
Cranial and paraspinal nerve tumors	16(2.75%)
Metastases to the CNS	13(2.23%)
Meningiomas	13(2.23%)
Mesenchymal, non-meningothelial tumors	9(1.55%)
Hematolymphoid tumors	6(1.03%)
Spinal cord tumor	3(0.52%)
Pineal tumors	1(0.17%)
Mixed	13(2.23%)
Unspecified	24(4.12%)
Top 10 retraction reasons by RWD (*N* = 582)	
Investigation by Journal/Publisher	276(47.42%)
Unreliable Results and/or Conclusions	235(40.38%)
Investigation by Third Party	207(35.57%)
Concerns/Issues about Data	181(31.10%)
Paper Mill^*^	178(30.58%)
Duplication of/in Image^*^	165(28.35%)
Concerns/Issues about Image	92(15.81%)
Compromised Peer Review^*^	79(13.57%)
Author Unresponsive	78(13.4%)
Concerns/Issues about Referencing/Attributions	77(13.23%)
New categories of retraction reasons (*N* = 582)	
Misconduct	469(80.58%)
Unverified misconduct	80(13.75%)
Error	15(2.58%)
Other	18(3.09%)
Number of authors (*N* = 582)	
1–3	110(18.90%)
4–6	246(42.27%)
7–9	147(25.26%)
≥ 10	79(13.57%)
Country distribution (*N* = 581)	
Single country	529(91.05%)
Internationally collaborative	52(8.95%)
China^#^	401(69.02%)
India^#^	47(8.09%)
USA^#^	68(11.7%)
Others^#^	65(11.19%)
JIF quartile (*N* = 572)	
Q1	186(32.52%)
Q2	236(41.26%)
Q3	129(22.55%)
Q4	21(3.67%)
JIF (*N* = 572)	
3–5	220(38.46%)
≤ 3	248(43.36%)
≥ 5	104(18.18%)

^*^ Reasons categorized in “Misconduct” group

^#^ means origin of first author

“*N*” represents the analytic sample size and varies across variables due to differential data availability. One paper without country information was excluded during the country distribution analysis (*N* = 581). Retractions published in Journals never indexed by Clarivate were removed from JIF and quartile analysis (*N* = 572)

**Table 2:** Recategorization of retraction reason

Re-categories	RWD retraction reasons
“Misconduct” category	Paper Mill, Duplication of/in Image, Compromised Peer Review, Computer-Aided Content or Computer-Generated Content, Euphemisms for Duplication, Euphemisms for Plagiarism, Lack of IRB/IACUC Approval and/or Compliance, Manipulation of Images, Misconduct by Author, Plagiarism of Image, Falsification/Fabrication of Data, Plagiarism of/in Article, Duplication of Data, Misconduct—Official Investigation(s) and/or Finding(s), Duplication of/in Article, False/Forged Authorship, Falsification/Fabrication of Image, Informed/Patient Consent—None/Withdrawn, Rogue Editor, Plagiarism of Text, Lack of Approval from Author, Ethical Violations by Author, Plagiarism of Data, Duplication of Text, Lack of Approval from Third Party, Conflict of Interest, Lack of Approval from Company/Institution, Ethical Violations by Company/Institution/Third Party, Falsification/Fabrication of Results, Manipulation of Results
“Unverified misconduct” category	Investigation by Journal/Publisher, Unreliable Results and/or Conclusions, Investigation by Third Party, Concerns/Issues about Data, Concerns/Issues about Image, Author Unresponsive, Concerns/Issues about Referencing/Attributions, Concerns/Issues about Results and/or Conclusions, Concerns/Issues about Peer Review, Original Data and/or Images not Provided and/or not Available, Unreliable Data, Investigation by Company/Institution, Objections by Author(s), Objections by Third Party, Concerns/Issues about Authorship/Affiliation, Concerns/Issues about Third Party Involvement, Results Not Reproducible, Concerns/Issues about Human Subject Welfare, Contamination of Cell Lines/Tissues, Concerns/Issues about Article, Breach of Policy by Author, Investigation by ORI, Concerns/Issues about Animal Welfare, Criminal Proceedings, Unreliable Image
“Error” category	Error in Analyses, Error in Data, Error in Results and/or Conclusions, Error in Image, Error in Methods, Error in Cell Lines/Tissues, Error in Materials, Error in Text, Error by Third Party
“Other” category	Upgrade/Update of Prior Notice(s), Date of Article and/or Notice Unknown, Notice—Limited or No Information, Removed, Copyright Claims, Duplication of Content through Error by Journal/Publisher, Error by Journal/Publisher, Miscommunication with/by Author, Miscommunication with/by Third Party, Notice—Unable to Access via current resources, Retract and Replace, Temporary Removal

The number of authors per paper ranged from 1 to 40. Retractions with 4 to 6 authors were the most common, comprising 42.27% (246/582) papers. Except for one retracted paper without country information, the remaining 581 papers had country information and involved 48 countries. Among these, 91.05% (529/581) papers were from a single country, and 8.95% (52/581) papers were collaborative works involving ≥ 2 countries. Regarding the country of the first author, China contributed the highest number of retracted papers, accounting for 69.02% (401/581), followed by USA and India with 11.70% (68/581) and 8.09% (47/581), respectively. The country distribution of first authors is shown in Additional file 3.

The median publication year for USA first-author papers was 2011 (IQR: 2005–2017), compared with 2019 (IQR: 2017–2021) for China and 2022 (IQR: 2020–2024) for India. Notably, 69.1% (47/68) of USA papers were published before 2015, compared with only 10.97% (44/401) for China and 10.6% (5/47) for India. The results showed that the USA subgroup had a significantly older publication-year distribution compared with China and India (*H* = 92.674, *P* < 0.001). Publication year and time to retraction were strongly negatively correlated across the full sample (*R* = –0.561, *P* < 0.001), with the strongest correlation observed in the USA subgroup (*R* = –0.784, *P* < 0.001), followed by India (*R* = –0.685, *P* < 0.001) and China (*R* = –0.520, *P* = 0.004).

The 582 retracted papers were published in 217 journals. There were 16 journals that had published ≥ 10 retracted papers each, accounting for 42.61% (248/582) of the total retracted publications. Among the 217 journals, 2024 JIF of 30 journals was unavailable in the 2024 JCR release: 19 were dropped, 1 was on hold, 6 were never indexed by Clarivate, 3 had ceased publication, and 1 was a conference proceedings journal. For the 23 journals that had been previously indexed (dropped, on hold, or ceased), their last reported JIF was retained for analysis; only the 7 never-indexed journals (10 papers) were excluded from JIF and quartile analyses, yielding an analytic sample of 572 for these metrics. These 30 journals collectively accounted for 19.93% (116/582) of retracted papers. The 19 dropped journals alone accounted for 16.15% (94/582) retracted papers.

The majority of retracted papers were basic research with 75.26% (438/582), followed by clinical research, 17.01% (99/582). Reviews/meta-analyses and commentaries together accounted for 45 papers.

Thirteen retracted papers were not indexed in Web of Science (11 were from the Misconduct category and 2 from the Other category; no articles were lost from the Unverified misconduct or Error categories), 54 had never been cited, and 515 were cited at least once, with a total of 12,151 citations. A total of 462 papers among these 515 retractions were cited at least once after retraction, and there were 35.34% (182/515) papers that received more citations after retraction. The maximum total citations, the maximum pre-retraction citations and the maximum post-retraction citations were 274, 272 and 113, respectively. The median pre-retraction citation, the median post-retraction citation and the median total citation were 5 (IQR: 1–17), 3 (IQR: 1–7) and 10 (IQR: 3–24), respectively. The longest retraction lag (time to retraction) was 9,973 days, the shortest was 0 days, the median retraction lag was 805.5 days (IQR: 423.25–1,711 days).

Annual citations in different periods were compared and the results were showed below. Significant differences across retraction reason categories were observed for annual pre-retraction citations (*H* = 9.822, *P* = 0.020) and total citations (*H* = 13.945, *P* = 0.003), but not for post-retraction citations (*H* = 6.215, *P* = 0.102) (Table 3). The unverified misconduct group had the highest median pre-retraction citations 2.57(0.8–4.92), and total citations 1.67(0.69–3.1). Regarding journal impact metrics, significant differences were found across JIF quartiles for annual pre-retraction citations (*H* = 24.461, *P* < 0.001), post-retraction citations (*H* = 9.364, *P* = 0.025) and total citations (*H* = 21.894, *P* < 0.001). Articles published in Q1 journals had the highest median pre-retraction citations 2.69(0.75–6.12), post-retraction citations 0.85(0.22–2.12) and total citations 1.87(0.62–4.1). For JIF groups (≤ 3, 3–5, ≥ 5), all three citation measures differed significantly (*H* = 34.076, 23.485, and 45.841, respectively, all *P* < 0.001). The JIF ≥ 5 group had the highest annual citation counts across all three time periods with pre-retraction citations 3.8(1.62–8.83), post-retraction citations 1.43(0.45–3.25) and total citations 2.98(1.35–5.74). By article type, all three citation measures also showed significant differences (*H* = 94.992, 60.406, and 111.579, respectively; all *P* < 0.001). Basic science articles had relatively high annual pre-retraction citations 2.61(1.08–4.96), post-retraction citations 0.91(0.39–1.88)and total citations 1.85(0.84–3.58), whereas commentaries had median citation counts of zero. For first-author country, significant differences were observed for annual pre-retraction citations (*H* = 11.852, *P* = 0.008) and total citations (*H* = 12.831, *P* = 0.005), but not for post-retraction citations (*H* = 3.465, *P* = 0.325). Articles with first authors from the USA had the highest median pre-retraction citations 2.56(0.87–4.94) and total citations 1.89(0.62–4.78).

**Table 3:** Analysis between variables and annual citation counts

Variables	Counts	Annual pre-retraction citations	Annual post-retraction citations	Annual total citations
Retraction reason categories (*N* = 569)				
Misconduct	458	2.04(0.6–4.24)	0.68(0.23–1.66)	1.36(0.54–3.12)
Unverified misconduct	80	2.57(0.8–4.92)	1.01(0.29–1.79)	1.67(0.69–3.1)
Error	15	2.52(0–3.8)	1.07(0.4–2.06)	1.1(0.35–2.32)
Other	16	0(0–1.53)	0.37(0–0.87)	0.39(0.11–0.99)
*H* value		9.822	6.215	13.945
*P* value		0.020	0.102	0.003
Article type (*N* = 569)				
Basic science	432	2.61(1.08–4.96)	0.91(0.39–1.88)	1.85(0.84–3.58)
Clinical research	97	0.49(0–1.38)	0.37(0–0.79)	0.48(0.1–1.09)
Commentary	18	0(0–0)	0(0–0)	0(0–0.44)
Review/meta-analysis	22	1.67(0.75–3.5)	0.6(0.24–0.94)	0.86(0.35–1.54)
*H* value		94.992	60.406	111.579
*P* value		< 0.001	< 0.001	< 0.001
First- author country (*N* = 568)				
China	398	2.16(0.75–4.23)	0.79(0.37–1.57)	1.39(0.59–2.56)
India	44	0.91(0–4.14)	0.48(0–2.88)	0.64(0–4.07)
USA	68	2.56(0.87–4.94)	0.54(0–2.03)	1.89(0.62–4.78)
Others	58	1.23(0–4.46)	0.52(0.11–1.59)	0.84(0.1–2.96)
*H* value		11.852	3.465	12.831
*P* value		0.008	0.325	0.005
JIF group (*N* = 563)				
3–5	219	2.18(0.51–4.28)	0.73(0.29–1.61)	1.39(0.51–2.53)
≤ 3	243	1.52(0–3.47)	0.54(0.19–1.3)	1.04(0.37–2.2)
≥ 5	101	3.8(1.62–8.83)	1.43(0.45–3.25)	2.98(1.35–5.74)
*H* value		34.076	23.485	45.841
*P* value		< 0.001	< 0.001	< 0.001
JIF quartile (*N* = 563)				
Q1	181	2.69(0.75–6.12)	0.85(0.22–2.12)	1.87(0.62–4.1)
Q2	234	2.04(0.6–4.41)	0.73(0.24–1.63)	1.4(0.49–2.65)
Q3	127	1.75(0.57–3.5)	0.75(0.37–1.34)	1.22(0.59–2.05)
Q4	21	0(0–1.18)	0.33(0–0.55)	0.39(0.17–0.61)
*H* value		24.461	9.364	21.894
*P* value		< 0.001	0.025	< 0.001

Normalized citations were expressed in median with interquartile range

“*N*” represents the analytic sample size and varies across variables due to differential data availability. Citation analysis was conducted only on articles that could be retrieved in the Web of Science (*N* = 569). Of the 13 articles not indexed in Web of Science, 11 were from the Misconduct category and 2 from the Other category; no articles were lost from the Unverified misconduct or Error categories. One paper without country information was excluded during the country distribution analysis (*N* = 568). Retractions published in Journals never indexed by Clarivate were removed from JIF and quartile analysis (*N* = 563)

Annual pre-retraction and post-retraction citations differed significantly within the majority of subgroups. Among articles retracted due to misconduct–related reason, annual pre-retraction citations were significantly higher than post-retraction citations (*P* < 0.001). Similar patterns were observed across basic science and review/meta-analysis subgroups, JIF groups, and first-author countries, except for some subgroups where differences were not statistically significant (e.g., India: *P* = 0.795; Q4 journals: *P* = 0.795). Pairwise comparison results of annual citation counts among groups are presented in Additional file 4.

Retraction lag varied significantly by retraction reason category (*H* = 28.50, *P* < 0.0001), with the misconduct group showing the longest median lag of 849(463–1843) days (Table 4). Significant differences were also observed across article types (*H* = 110.70, *P* < 0.001); basic science articles had the longest median lag of 1025(557–1973) days, while commentaries had the shortest 130(118.5–139) days.

**Table 4:** Analysis between variables and time to retraction

Variables	Counts	Time to retraction	*H* value	*P* value
Retraction reason categories (*N* = 582)				
Misconduct	469	849(463–1843)	28.501	< 0.001
Unverified misconduct	80	734(372.5–1317)		
Error	15	212(124–652)		
Other	18	213(19–1035)		
Article type (*N* = 582)				
Basic Science	438	1025(557–1973)	110.696	< 0.001
Clinical Research	99	420(236–712)		
Commentary	20	130(118.5–139)		
Review/Meta-analysis	25	609(298–792)		
Number of authors group (*N* = 582)				
1—3	110	560(181–1063)	35.227	< 0.001
4—6	246	754(395–1511)		
7—9	147	1035(540–2275)		
≥ 10	79	1211(575–2193)		
First-author country (*N* = 581)				
China	401	805(455–1495)	61.386	< 0.001
India	47	449(132–700)		
USA	68	3416.5(915.5–5492.5)		
Others	65	708(273–1449)		
JIF group (*N* = 572)				
3—5	220	776.5(473–1523.5)	13.970	0.001
≤ 3	248	725.5(321–1524)		
≥ 5	104	1207.5(461–2356.5)		
JIF quartile (*N* = 572)				
Q1	186	988.5(393–1961)	12.055	0.007
Q2	236	808(479.5–1815)		
Q3	129	673(365–1262)		
Q4	21	583(236–929)		

Time to retraction was expressed in median with interquartile range

“*N*” represents the analytic sample size and varies across variables due to differential data availability. One paper without country information was excluded during the country distribution analysis (*N* = 581). Retractions published in Journals never indexed by Clarivate were removed from JIF and quartile analysis (*N* = 572)

Retraction lag increased with the number of authors (*H* = 35.23, *P* < 0.001), ranging from a median of 560 (181–1063) days for articles with 1–3 authors to 1,211 (575–2193) days for those with ≥ 10 authors. Finally, significant differences were found across first‑author countries (*H* = 61.386, *P* < 0.001). Articles with first authors from USA had the longest median retraction lag of 3416.5 (915.5–5492.5) days, while India had the shortest 449(132–700) days.

Pairwise comparisons of retraction lag revealed specific group differences underlying the overall Kruskal–Wallis findings. For example, “Misconduct” group showed significantly longer retraction lag than both “Error” group (*P* < 0.0001) and “Other” group (*P* = 0.001), but the difference between “Misconduct” and “Unverified misconduct” did not reach statistical significance (*P* = 0.079). Pairwise comparison results of retraction lag among groups are presented in Additional file 5.

## Discussion

This study provides the first systematic bibliometric analysis of retracted publications in CNS tumor research. The number of retracted CNS tumor publications has exhibited a strong upward trend over time.

Notably, retractions due to misconduct accounted for 80.58% (469/582), suggesting that research misconduct rather than honest errors may be associated with the majority of retractions in this field. Although different methods of categories were used, misconduct was defined to be the main reason for retraction in many fields [19]. The proportion of retractions attributable to clinical research in our dataset is 17.01% (99/582) and substantially lower than that reported in neurosurgery retractions (55% clinical neurosurgery) and retracted spine literature (56% clinical studies), highlighting potential differences in research integrity patterns across subspecialties [20, 21].

One of the most concerning findings is that 89.71% (462/515) of retracted CNS tumor articles continued to receive citations after retraction, and 35.34% (182/515) actually received more citations after retraction than before. This phenomenon was also observed in previous studies of other fields [4, 22]. Articles that cited retracted publications exhibited higher risk of subsequent retraction [4]. Notably, Clarivate will no longer include citations to and from retracted articles in JIF calculations from 2025 [18]. This policy change, though beneficial to a certain degree, does not resolve the deeper issue: retracted articles continue to be cited inappropriately as valid evidence.

Systematic analyses have revealed that retraction notices themselves are often delayed, incomplete, or difficult to access, which is associated with the persistence of citations to retracted work [23, 24]. This persistent post-retraction circulation of flawed evidence suggests that current mechanisms for retraction status across indexing platforms are insufficient to alert researchers to the retracted status of affected articles.

In this study, "Paper Mill", "Duplication of/in Image", and "Compromised Peer Review" were the top three misconduct reasons, accounting for 30.58% (178/582), 28.35% (165/582) and 13.57% (79/582), respectively. The shift from traditional forms such as plagiarism to more sophisticated forms is consistent with the evolving complexity of fraudulent practices [25]. A rapid scoping review of paper mills identified increasingly complex processes involving fabricated images and fake results [26, 27]. Image duplication or manipulation has also become one of the most frequently detected issues leading to retraction [28]. Compromised peer review has emerged as another recurrent concern in recent years and has caused a large number of retractions [5, 11, 29].

"Investigation by Journal/Publisher" was the single most common retraction reason in this study accounting for 47.42% (276/582). This points to a clear shortcoming in pre-publication peer review, while also showing that editorial scrutiny after publication has become an important line of defense [30]. The COPE retraction guidelines take the same view, assigning journals the main responsibility for investigating misconduct allegations [2].

In terms of absolute numbers rather than retraction rates, China was the leading first-author country with 69.02% (401/581), followed by the USA 11.70% (68/581), and India 8.09% (47/581). Similar patterns have been observed in other retraction analyses [4, 10, 28, 31]. It may be associated with the high volume of research output in these countries. In recent years, the Chinese government has enacted numerous policies and regulations concerning scientific research integrity and reflects China's recognition of this challenge and its commitment to strengthening research integrity oversight [32]. Despite these policy efforts, the persistently high proportion of retractions from Chinese authors suggests that policy implementation at the institutional level requires further reinforcement [33].

In India, the rapid growth in research output has been accompanied by emerging challenges in research oversight infrastructure, with retractions frequently initiated by journals rather than institutional investigations. It is important to note that without denominator data (total publications per country), these figures represent absolute counts rather than retraction rates, and a country with a large research output may have more retractions simply due to volume.

Importantly, 91.05% (529/581) of retracted articles were single-country publications. This finding shows that most retracted CNS tumor publications were single-country publications. However, whether international collaboration serves as a protective factor against misconduct cannot be determined from the present data, as collaboration patterns are influenced by multiple confounding factors including research field, journal policies, funding sources, and publication volume [30, 34].

The finding that 30 journals lacked 2024 JIF, including 19 journals dropped by Clarivate, is particularly concerning. Among the top 15 journals with the highest retraction counts, five were dropped journals. This pattern is associated with journals that have been dropped or suppressed, which may reflect compromised editorial standards; however, whether these journals actively facilitated the publication of flawed research cannot be determined from this cross-sectional, count-based design. The observation that journals are dropped or suppressed is associated with patterns of systematic manipulation of the publication process, including compromised peer review, the operation of paper mills, and large-scale citation manipulation [35]. For CNS tumor research, the presence of such journals in the publishing ecosystem is concerning. Researchers may cite articles unknowingly from these compromised sources which is associated with the dissemination of unreliable data.

The median time to retraction was 805.5 days (approximately 2.2 years). Several factors may be associated with these retractions delays, including non-standardized retraction procedures, complicated investigation process,difficulties in reaching consensus among authors or institutions, incomplete retraction notices and inconsistent indexing across different databases [36].

The "misconduct" group exhibited the longest median retraction lag (849 days), likely reflecting the time required for investigations by journals, publishers, and institutions. Basic science articles showed the longest retraction lag, nearly 3 years, while commentaries had the shortest (130 days). This disparity is consistent with the hypothesis that the nature of basic research and investigations often require extensive review of raw data, image forensics, and institutional inquiries.

The positive association between author count and retraction lag (from 560 days for 1–3 authors to 1,211 days for ≥ 10 authors) was also observed in previous studies and is consistent with the hypothesis that larger author teams complicate investigation logistics [9, 34].

The notably longer retraction lag for USA first-author papers (median 3,416.5 days, ~ 9.4 years) reflects the composition of this subgroup, which includes a substantial proportion of older publications (median publication year 2011; 69.1% published before 2015) that may have extended investigation timelines. The strong negative correlation between publication year and retraction lag (*R* = –0.784, *P* < 0.001) confirms that the long lag is concentrated among older publications rather than representing a systematic difference in national retraction processes.

Retraction is only the first step for correction. The continuous citation or utilization of retracted papers in subsequent research, which creates a contamination chain of flawed evidence, is the critical issue that demands attention [37–39]. In neuro-oncology, where experimental findings may inform glioma grading, treatment selection, imaging interpretation, and prognostic assessment, persistent citation of retracted studies can affect how later evidence is framed and interpreted.

## Limitations

Several limitations should be noted. First, although RWD is comprehensive, it may still have a certain degree of bias because it cannot cover all retraction information. Second, our search strategy relied on title-level keyword screening which may have missed some relevant articles. Third, citation data were retrieved exclusively from Web of Science, with limited coverage of non-English journals. Fourth, classification of retraction reasons and article type for each retraction requires subjective judgment and may vary across different studies. Fifth, the country of origin was determined solely by the first author's affiliation, which may not fully reflect international collaborative research. Sixth, this study analyzed only retracted articles without evaluating the proportion of retractions relative to total publication output in each category; without denominator data, our findings describe the absolute distribution rather than the relative risk of retraction. Finally, although normalized citation counts (citations per year) were used to adjust for unequal exposure times, citation counts may still be influenced by secular trends in citation practices and database coverage over time.

## Clinical recommendations

The persistence and high rate of post-retraction citations in CNS tumor field raise concerns about evidence-based patient care. Clinicians and principal investigators are strongly advised to check the retraction status of articles before citing them, via databases such as Retraction Watch, PubMed, or Web of Science.

## Conclusions

This study provides the first systematic bibliometric analysis of retracted publications in CNS tumor research. The number of retractions in this field has increased substantially over time, with misconduct accounting for the overwhelming majority (80.58%, 469/582) of retractions. The persistent post-retraction citation of flawed articles is concerning for evidence-based neuro-oncology and patient safety. The median retraction lag of over 2 years allows invalid evidence to accumulate citations and influence subsequent research before correction. These findings reveal that the current mechanisms for identifying, processing, and communicating retractions are insufficient to prevent the continued dissemination of flawed research in neuro-oncology, as evidenced by the median retraction lag of over 2 years and the persistent post-retraction citation of 89.71% (462/515) of retracted articles.

## Supplementary Information

Additional file 1: Search terms for CNS tumors retractions in the Retraction Watch Database based on the 2021 WHO Classification of Tumors of the CNS (5th edition). A complete bilingual list of 12 categories of CNS tumor search terms used to screen the Crossref/Retraction Watch Database for identifying CNS tumor-related retractions.

Additional file 2: Retractions categorized by year of original publication and retraction. Annual distribution of original publications and retractions from 1975 to 2025, showing the temporal trends of publication and retraction activities in the field of CNS tumors.

Additional file 3: Distribution of retracted CNS tumor publications by first-author country. The geographical distribution of 582 retractions in CNS tumor research.

Additional file 4: The differences among groups regarding annual pre-retraction citations, post-retraction citations and total citation counts. Pairwise comparison *P* values for pre-retraction citations vs post-retraction citations, pre-retraction citations vs total citations, and post-retraction citations vs total citations, stratified by retraction reason, article type, first-author country, JIF group, and JIF quartile.

Additional file 5: The differences among subgroups regarding time to retraction. Pairwise comparison *P* values for retraction lag across retraction reason categories, article types, author number groups, first-author countries, JIF groups, and JIF quartiles.

## Data Availability

The raw data analysis in this study was downloaded from the Crossref/Retraction Watch database which was open to public. The datasets generated and/or analyzed during the current study are available from the corresponding author on reasonable request (Hao Wang, cmu990103@163.com).

## Abbreviations

JIF: Journal impact factor
RWD: Retraction Watch database
CNS: Central nervous system
