# Supplementary material for: Retractions of publications on central nervous system tumors based on the Retraction Watch Database
Source: Chin Neurosurg J. 2026 Aug 10;12:24. doi: 10.1186/s41016-026-00444-8 (PMC13455306; doi:10.1186/s41016-026-00444-8)
Supplement: Supplementary file 1 [file 41016_2026_444_MOESM1_ESM.docx]

| **Categories** | **Search Terms** |
| --- | --- |
| **Gliomas, glioneuronal tumors, and neuronal tumors** | Gliomas; Astrocytoma; Oligodendroglioma; Glioblastoma; Diffuse astrocytoma; Angiocentric glioma; Polymorphous low-grade neuroepithelial tumor; Diffuse low-grade glioma; Diffuse midline glioma; Diffuse hemispheric glioma; Infant-type hemispheric glioma; Pilocytic astrocytoma; High-grade astrocytoma with piloid features; Pleomorphic xanthoastrocytoma; Subependymal giant cell astrocytoma; Chordoid glioma; Astroblastoma; Ganglioglioma; Desmoplastic infantile ganglioglioma; Dysembryoplastic neuroepithelial tumor; Diffuse glioneuronal tumor; Papillary glioneuronal tumor; Rosette-forming glioneuronal tumor; Myxoid glioneuronal tumor; Diffuse leptomeningeal glioneuronal tumor; Gangliocytoma; Multinodular and vacuolating neuronal tumor; Dysplastic cerebellar gangliocytoma; Central neurocytoma; Extraventricular neurocytoma; Cerebellar liponeurocytoma; Ependymoma; Supratentorial ependymoma; Posterior fossa ependymoma; Spinal ependymoma; Spinal ependymoma MYCN-amplified; Myxopapillary ependymoma; Subependymoma; |
| **Choroid plexus tumors** | Choroid plexus papilloma; Atypical choroid plexus papilloma; Choroid plexus carcinoma |
| **Embryonal tumors** | Medulloblastoma; Atypical teratoid rhabdoid tumor; Cribriform neuroepithelial tumor; Embryonal tumor with multilayered rosettes; CNS neuroblastoma; CNS tumor with BCOR internal tandem duplication; CNS embryonal tumor |
| **Pineal tumors** | Pineocytoma; Pineal parenchymal tumor of intermediate differentiation; Pineoblastoma; Papillary tumor of the pineal region; Desmoplastic myxoid tumor of the pineal region |
| **Cranial and paraspinal nerve tumors** | Schwannoma; Neurofibroma; Perineurioma; Hybrid nerve sheath tumor; Malignant melanotic nerve sheath tumor; Malignant peripheral nerve sheath tumor; Paraganglioma |
| **Meningiomas** | Meningioma |
| **Mesenchymal, non-meningothelial tumors** | Solitary fibrous tumor; Hemangioblastoma; Rhabdomyosarcoma; Intracranial mesenchymal tumor; CIC-rearranged sarcoma; Primary intracranial sarcoma; Ewing sarcoma; Mesenchymal chondrosarcoma; Chondrosarcoma; Chordoma; Meningeal melanocytoma; Meningeal melanoma |
| **Melanocytic tumors** | Meningeal melanocytosis; Meningeal melanomatosis; Meningeal melanocytoma; Meningeal melanoma |
| **Hematolymphoid tumors** | Primary diffuse large B-cell lymphoma of the CNS; Immunodeficiency-associated CNS lymphoma; Lymphomatoid granulomatosis; Intravascular large B-cell lymphoma; MALT lymphoma of the dura; Anaplastic large cell lymphoma; Erdheim-Chester disease; Rosai-Dorfman disease; Juvenile xanthogranuloma; Langerhans cell histiocytosis; Histiocytic sarcoma |
| **Germ cell tumors** | Teratoma; Germinoma; Embryonal carcinoma; Yolk sac tumor; Choriocarcinoma; Mixed germ cell tumor |
| **Tumors of the sellar region** | Craniopharyngioma; Adamantinomatous craniopharyngioma; Papillary craniopharyngioma; Pituicytoma; Granular cell tumor of the sellar region; Spindle cell oncocytoma; Pituitary adenoma; PitNET; Pituitary blastoma |
| **Metastases to the CNS** | Metastases; Brain metastasis; Meningeal metastasis; Metastases to the brain; Metastases to spinal cord parenchyma; Metastases to the meninges |

**Additional file 1.** Search terms for CNS tumors retractions in RWD based on the 2021 WHO Classification of Tumors of the CNS (5th edition)
