# Supplementary material for: Retractions of publications on central nervous system tumors based on the Retraction Watch Database
Source: Chin Neurosurg J. 2026 Aug 10;12:24. doi: 10.1186/s41016-026-00444-8 (PMC13455306; doi:10.1186/s41016-026-00444-8)
Supplement: Supplementary file 2 [file 41016_2026_444_MOESM2_ESM.docx]

**Additional file 2.** Retractions categorized by year of original publication and retraction

| **Year** | **Publications** | **Retractions** |
| --- | --- | --- |
| 1975 | 2 | 0 |
| 1976 | 1 | 0 |
| 1977 | 0 | 3 |
| 1978 | 0 | 0 |
| 1979 | 0 | 0 |
| 1980 | 0 | 0 |
| 1981 | 0 | 0 |
| 1982 | 0 | 0 |
| 1983 | 0 | 0 |
| 1984 | 0 | 0 |
| 1985 | 0 | 0 |
| 1986 | 0 | 0 |
| 1987 | 0 | 0 |
| 1988 | 0 | 0 |
| 1989 | 0 | 0 |
| 1990 | 0 | 0 |
| 1991 | 0 | 0 |
| 1992 | 0 | 0 |
| 1993 | 1 | 0 |
| 1994 | 0 | 0 |
| 1995 | 2 | 0 |
| 1996 | 0 | 1 |
| 1997 | 1 | 0 |
| 1998 | 1 | 0 |
| 1999 | 0 | 1 |
| 2000 | 0 | 0 |
| 2001 | 6 | 0 |
| 2002 | 5 | 0 |
| 2003 | 3 | 1 |
| 2004 | 5 | 1 |
| 2005 | 2 | 0 |
| 2006 | 2 | 1 |
| 2007 | 3 | 0 |
| 2008 | 2 | 2 |
| 2009 | 8 | 1 |
| 2010 | 14 | 4 |
| 2011 | 18 | 5 |
| 2012 | 9 | 2 |
| 2013 | 16 | 9 |
| 2014 | 27 | 3 |
| 2015 | 33 | 12 |
| 2016 | 31 | 17 |
| 2017 | 50 | 36 |
| 2018 | 41 | 12 |
| 2019 | 74 | 11 |
| 2020 | 61 | 44 |
| 2021 | 41 | 88 |
| 2022 | 82 | 68 |
| 2023 | 16 | 130 |
| 2024 | 24 | 72 |
| 2025 | 1 | 58 |
