# Supplementary material for: Retractions of publications on central nervous system tumors based on the Retraction Watch Database
Source: Chin Neurosurg J. 2026 Aug 10;12:24. doi: 10.1186/s41016-026-00444-8 (PMC13455306; doi:10.1186/s41016-026-00444-8)
Supplement: Supplementary file 3 [file 41016_2026_444_MOESM3_ESM.docx]

**Additional file 3.** Distribution of retracted CNS tumor publications by first-author country

| First-author country | Number of retractions |
| --- | --- |
| China | 401 |
| USA | 68 |
| India | 47 |
| Italy | 6 |
| Japan | 6 |
| Iran | 5 |
| South Korea | 5 |
| Germany | 4 |
| Netherlands | 4 |
| Bangladesh | 3 |
| Brazil | 3 |
| Singapore | 3 |
| Canada | 2 |
| Colombia | 2 |
| Russia | 2 |
| Saudi Arabia | 2 |
| Switzerland | 2 |
| Turkey | 2 |
| United Kingdom | 2 |
| Belgium | 1 |
| Chile | 1 |
| Ireland | 1 |
| Israel | 1 |
| Malaysia | 1 |
| Mexico | 1 |
| Morocco | 1 |
| Nigeria | 1 |
| Pakistan | 1 |
| Romania | 1 |
| Spain | 1 |
| Sweden | 1 |
| Unknown | 1 |
