# Supplementary material for: Retractions of publications on central nervous system tumors based on the Retraction Watch Database
Source: Chin Neurosurg J. 2026 Aug 10;12:24. doi: 10.1186/s41016-026-00444-8 (PMC13455306; doi:10.1186/s41016-026-00444-8)
Supplement: Supplementary file 4 [file 41016_2026_444_MOESM4_ESM.docx]

| **Variables** | **P value  (annual pre-retraction citations vs annual post-retraction citations)** | **P value  (annual pre-retraction citations vs annual total citations)** | **P value  (annual post-retraction citations vs annual total citations)** |
| --- | --- | --- | --- |
| **Retraction reason categories (N = 569)** | | | |
| Misconduct | <0.001 | 0.010 | <0.001 |
| Unverified misconduct | 0.001 | 0.182 | 0.005 |
| Error | 0.586 | 0.615 | 0.868 |
| Other | 0.694 | 0.471 | 0.804 |
| **Article type (N = 569)** | | | |
| Basic Science | <0.001 | <0.001 | <0.001 |
| Clinical Research | 0.964 | 0.319 | 0.229 |
| Commentary | 0.772 | 0.200 | 0.318 |
| Review/ Meta-analysis | 0.006 | 0.072 | 0.185 |
| **First-author country (N = 568)** | | | |
| China | <0.001 | 0.001 | <0.001 |
| India | 0.795 | 0.804 | 0.507 |
| USA | <0.001 | 0.470 | <0.001 |
| others | 0.620 | 0.665 | 0.298 |
| **JIF group (N = 563)** | | | |
| 3—5 | <0.001 | 0.040 | <0.001 |
| ≤3 | <0.001 | 0.077 | <0.001 |
| ≥5 | <0.001 | 0.178 | <0.001 |
| **JIF quartile (N = 563)** | | | |
| Q1 | <0.001 | 0.067 | <0.001 |
| Q2 | <0.001 | 0.036 | <0.001 |
| Q3 | <0.001 | 0.087 | 0.001 |
| Q4 | 0.795 | 0.261 | 0.384 |

**Additional file 4.** The differences among groups regarding annual pre-retraction citations, post-retraction citations and total citation counts

Note: “N” represents the analytic sample size and varies across variables due to differential data availability. Citation analysis was conducted only on articles that could be retrieved in the Web of Science (N=569). Of the 13 articles not indexed in Web of Science, 11 were from the Misconduct category and 2 from the Other category; no articles were lost from the Unverified misconduct or Error categories. One paper without country information was excluded during the country distribution analysis (N=568). Retractions published in Journals never indexed by Clarivate were removed from JIF and quartile analysis (N=563).
