# Supplementary material for: Retractions of publications on central nervous system tumors based on the Retraction Watch Database
Source: Chin Neurosurg J. 2026 Aug 10;12:24. doi: 10.1186/s41016-026-00444-8 (PMC13455306; doi:10.1186/s41016-026-00444-8)
Supplement: Supplementary file 5 [file 41016_2026_444_MOESM5_ESM.docx]

**Additional file 5.** The differences among subgroups regarding time to retraction

| **Variables** | **P value** |
| --- | --- |
| **Retraction reason categories (N = 582)** | |
| Error vs Other | 0.871 |
| Error vs Misconduct | <0.001 |
| Error vs Unverified misconduct | 0.001 |
| Misconduct vs Other | 0.001 |
| Unverified misconduct vs Other | 0.008 |
| Unverified misconduct vs Misconduct | 0.079 |
| **Article type (N=582)** | |
| Basic Science vs Clinical Research | <0.001 |
| Basic Science vs Commentary | <0.001 |
| Basic Science vs Review/Meta-analysis | 0.002 |
| Commentary vs Clinical Research | <0.001 |
| Review/Meta-analysis vs Clinical Research | 0.171 |
| Review/Meta-analysis vs Commentary | <0.001 |
| **Number of authors group (N = 582)** | |
| 1–3 vs 4–6 | 0.004 |
| 1–3 vs 7–9 | <0.001 |
| 1–3 vs ≥10 | <0.001 |
| 4–6 vs 7–9 | 0.001 |
| 4–6 vs ≥10 | 0.006 |
| ≥10 vs 7–9 | 0.702 |
| **First**-author country **(N = 581)** | |
| China vs India | <0.001 |
| China vs others | 0.426 |
| China vs USA | <0.001 |
| others vs India | 0.002 |
| USA vs India | <0.001 |
| USA vs others | <0.001 |
| **JIF group (N = 572)** | |
| 3–5 vs ≥5 | 0.005 |
| 3–5 vs ≤3 | 0.184 |
| ≥5 vs ≤3 | <0.001 |
| **JIF quartile (N = 572)** | |
| Q1 vs Q2 | 0.784 |
| Q1 vs Q3 | 0.019 |
| Q1 vs Q4 | 0.032 |
| Q2 vs Q3 | 0.009 |
| Q2 vs Q4 | 0.017 |
| Q3 vs Q4 | 0.196 |

Note: “N” represents the analytic sample size and varies across variables due to differential data availability. One paper without country information was excluded during the country distribution analysis (N=581). Retractions published in Journals never indexed by Clarivate were removed from JIF and quartile analysis (N=572).
